# Correction: The role of renal and liver function in clinical ctDNA testing

**DOI:** 10.1371/journal.pone.0330539

**Published:** 2025-08-18

**Authors:** The PLOS ONE Staff

## Notice of Republication

This article was republished on August 4, 2025, to correct an error in the XML. A corrupted version of Figures 1 and 3 were published, in which important details of Figures 1C and 3C were obscured. The publisher apologizes for this error.
