# Supplementary material for: The role of renal and liver function in clinical ctDNA testing
Source: PLoS One. 2025 Feb 25;20(2):e0319194. doi: 10.1371/journal.pone.0319194 (PMC11856342; doi:10.1371/journal.pone.0319194)
Supplement: S1 File — (DOCX) [file pone.0319194.s004.docx]

# S1 file. Methodology for eGFR calculations. Method code: DNK35302

eGFR / 1,73m²(CKD-EPI) is automatically provided for patients when P-creatinine is requisitioned on patients > 17 years of age

eGFR is calculated via the CKD-EPI formula without race correction as recommended in the report by the Danish society of nephrologists, Danish pediatric society and Danish society for clinical biochemistry and Danish society for clinical biochemistry: chronic kidney disease: analysis methods and clinical evaluation

# Formulas:

# Female


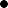
 P-Creatinine ≤62 µmol/l:

$$eGFR=144\cdot\left( \frac{\mathrm{creatinine}}{0.7\cdot88.4} \right)^{-0.329}\cdot{0.993}^{\mathrm{age}}$$

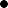
 P-Creatinine > 62 µmol/l:

$$eGFR=144\cdot\left( \frac{\mathrm{creatinine}}{0.7\cdot88.4} \right)^{-1.209}\cdot{0.993}^{\mathrm{age}}$$

# Male

P-Creatinine ≤80 µmol/l:

$$eGFR=141\cdot\left( \frac{\mathrm{creatinine}}{0.9\cdot88.4} \right)^{-0.411}\cdot{0.993}^{\mathrm{age}}$$

P-Creatinine > 80 µmol/l:

$$eGFR=141\cdot\left( \frac{\mathrm{creatinine}}{0.9\cdot88.4} \right)^{-0.411}\cdot{0.993}^{\mathrm{age}}$$

The above formulas have been acquired from the department of biochemistry in Aarhus university hospital’s website: <https://www.fagperson.auh.dk/afdelinger/blodprover-og-biokemi/analysefortegnelsen/hospital/>. Last accessed 27-09-2024.
