# Supplementary material for: The role of renal and liver function in clinical ctDNA testing
Source: PLoS One. 2025 Feb 25;20(2):e0319194. doi: 10.1371/journal.pone.0319194 (PMC11856342; doi:10.1371/journal.pone.0319194)
Supplement: S2 File — (DOCX) [file pone.0319194.s005.docx]

**Renal and liver function effect on cfDNA results in the postoperative setting**

**Introduction**In the main article, we report on the preoperative correlation between laboratory measurements of renal/liver function biomarkers and cell-free DNA (cfDNA) and circulating tumor DNA (ctDNA) levels. There is also great interest in exploring the postoperative correlation between these markers, therefore we provide such an analysis as supporting information. We note however, that our study was not designed with postoperative analysis in mind. Consequently, the postoperative results, in contrast to the preoperative results, are affected by numerous limitations.

For example, the consequences of the trauma induced by surgery. We have previously shown that surgical trauma is associated with a temporary elevation in the level of normal DNA in the circulation[1]. This elevation persists for up to 4 weeks after surgery [1] and in this window any correlation between cfDNA and renal/liver laboratory biomarkers will be obscured. Obviously, surgery also impacts the ctDNA level. Most patients are cured by surgery and hence will have no ctDNA in the blood postoperatively. Also, adjuvant chemotherapy (ACT) obscures the correlation between renal/liver function and cfDNA/ctDNA. The purpose of ACT is to lower, and potentially eliminate, any residual disease in the patients. Hence, ACT will also affect the ctDNA level. ACT also impacts the hematopoietic cell turnover, and thereby the level of normal DNA in the blood. ACT is also likely to affect renal and liver function, and thereby cfDNA clearance. Consequently, to avoid the potential confounding effects of the surgical trauma and ACT, the measurements of cfDNA/ctDNA/renal/liver biomarkers must be performed in the narrow window between week four and start of ACT.

Furthermore, preoperatively the renal/liver biomarkers serve as a baseline to evaluate the fitness of a patient for CT-imaging and surgery, consequently they are measured on many patients. This contrasts the postoperative setting, where the biomarkers mainly are measured if there is a clinical indication. Therefore, postoperatively standard of care renal/liver biomarker measurements are only available for a minor subset of patients, weakening the statistical power to assess correlation. Furthermore, if a correlation between renal/liver and cfDNA/ctDNA markers is observed postoperatively, it is vulnerable to confounding by indication.

As the renal/liver biomarker results are generated due to clinical indications, they are monitored closely. The aim being to treat dangerous conditions with quick clinical intervention. In multiple patients we observe oscillating renal/liver biomarker levels indicative of multiple interventions. An example of 6 patients’ serial potassium samples is shown below (S3 Fig). This increases the need for the cfDNA and kidney/liver measurements to be done close to each other. If a renal/liver function intervention is made between the time points of the renal/liver and cfDNA measurements, there is a risk it will obscure any potentially correlation.

**S3 Fig** line plots showing potassium levels for six patients in the cohort. Each line is a single patient’s potassium levels from day of surgery until 60 days after. The red dotted lines signify upper and lower reference range bounds.

With the above challenges in mind, we have conducted a postoperative analysis of the correlation between renal/liver and cfDNA biomarkers.

**Methods**

The postoperative methodology follows the same methods as described in sections 2.1 through 2.4 of the main article. Postoperative laboratory test results were matched with postoperative cfDNA measurements. Only cfDNA samples collected in the timeframe of 28 to 57 days after surgery were included. Laboratory biomarkers were matched to cfDNA samples if the laboratory markers were measured no more than five days after the cfDNA sample. All samples, both cfDNA and laboratory, were only included if they were collected prior to the initiation of ACT. If there were multiple valid matches for the same laboratory marker, the one closest in time was used. Low renal function was defined as in section 2.6 of the main article.
To investigate the relationship between liver and renal laboratory test levels and postoperative cfDNA level, a log-log linear regression was used. The association between renal function and postoperative cfDNA level was analyzed with a binomial logistic regression.

| **Characteristic** | **N = 33^1^** |
| --- | --- |
| Age | 68 (58, 77) |
| Sex |  |
| Female | 23 (70%) |
| Male | 10 (30%) |
| pT stage |  |
| pT2 | 4 (12%) |
| pT3 | 23 (70%) |
| pT4 | 6 (18%) |
| pN stage |  |
| pN0 | 15 (45%) |
| pN1 | 11 (33%) |
| pN2 | 7 (21%) |
| Location |  |
| Left side | 21 (64%) |
| Right side | 11 (33%) |
| Rectum | 1 (3.0%) |
| ctDNA call |  |
| Negative | 30 (91%) |
| Positive | 3 (9.1%) |
| ^1^ **Median (IQR); n (%)** |  |

**Results**

Of the 846 patients included in the main article, 253 patients had a postoperative cfDNA measurement 28 to 57 days after surgery and before start of ACT. Of these, 33 patients had renal/liver laboratory biomarkers measured in a time window from cfDNA within five days after the cfDNA sample date (S4 Fig). Cohort characteristics can be seen in S8 table.

**S4 Fig** Cohort funnel showing the number of excluded patients for the postoperative analysis and final sample availability.

To investigate if postoperative cfDNA correlated with renal and liver function, we analyzed whether the biomarkers of renal and liver function were correlated cfDNA level (S5 Fig). We additionally analyzed whether patients with normal or low renal function differed in their cfDNA levels (S6 Fig). None of the biomarkers were significantly associated with cfDNA level. Full regression results are available in the regression tables section of this document.

**S8 Table: Clinical characteristics of the postoperative cohort**

**S5 Fig** Scatterplots comparing postoperative cfDNA level to renal and liver function laboratory test results. Dots are colored for the reference range categories. A log-log regression was used to analyze the relationship between the laboratory test results and the cfDNA level. For eGFR, a result above 90 mL/min/1.73m2 was treated as 90 mL/min/1.73m2. All regression models were adjusted for patient age at date of ctDNA sampling, and the pT and pN categories of the tumor. In all analyses, the false discovery rate was controlled using the Benjamini-Hochberg method. A significant result was defined as p < 0.05 after Benjamini-Hochberg adjustment. Note that different patients can have different reference ranges depending on age, sex, and region of origin. Therefore, the same laboratory result can be categorized in different reference range categories.

**S6 Fig** Box plot showing the postoperative cfDNA level in patients with low renal function and patients with normal renal function. The association was assessed using binomial logistic regression. The statistical analysis was adjusted for pT stage and pN stage as well as age at sample date.

**Discussion and limitations**

The analyses of the postoperative renal/liver and cfDNA biomarker measurements did not show any correlation. The results should be interpreted with caution, as the sample size was limited and there is great risk of indication bias.

The postoperative analyses have several limitations as outlined above. Despite the inclusion of more than 800 CRC patients in our study, the high cure rate resulted in a very low incidence of post-operative ctDNA detections. This prohibited analysis of correlation between ctDNA levels and renal/liver function.

**Supporting References**

1. Henriksen TV, Reinert T, Christensen E, Sethi H, Birkenkamp-Demtröder K, Gögenur M, et al. The effect of surgical trauma on circulating free DNA levels in cancer patients—implications for studies of circulating tumor DNA. Molecular Oncology. 2020;14(8):1670-9.

2. Nors J, Iversen LH, Erichsen R, Gotschalck KA, Andersen CL. Incidence of Recurrence and Time to Recurrence in Stage I to III Colorectal Cancer: A Nationwide Danish Cohort Study. JAMA Oncology. 2024;10(1):54-62.

**Supporting information regression Tables**

| **Sodium** |  |  |  |  |  |
| --- | --- | --- | --- | --- | --- |
| Term | Slope | 2.5 % | 97.5 % | Standard error | p value |
| (Intercept) | 0 | 0 | 39564535681 | 18.61454 | 0.461041808 |
| log10 (Laboratory test result) | 2994.30357 | 0.00006 | 1.44145E+11 | 8.58912 | 0.360285396 |
| stage_pT3 | 0.83119 | 0.4918 | 1.40478 | 0.25481 | 0.474791681 |
| stage_pT4 | 0.87747 | 0.46838 | 1.64385 | 0.3048 | 0.671702104 |
| stage_pN1 | 0.92454 | 0.6028 | 1.41801 | 0.20767 | 0.70877289 |
| stage_pN2 | 0.69477 | 0.42692 | 1.13067 | 0.23645 | 0.136083542 |
| Age_At_Sample_date | 1.00636 | 0.99032 | 1.02267 | 0.0078 | 0.424033003 |
| R = 0.03591826 |  |  |  |  |  |
|  |  |  |  |  |  |
| **Potassium** |  |  |  |  |  |
| Term | Slope | 2.5 % | 97.5 % | Standard error | p value |
| (Intercept) | 254.90948 | 23.84038 | 2725.57887 | 1.15051 | 6.0019E-05 |
| log10 (Laboratory test result) | 0.01624 | 0.00027 | 0.98326 | 1.99233 | 0.049133857 |
| stage_pT3 | 0.79019 | 0.48651 | 1.28344 | 0.2355 | 0.326938809 |
| stage_pT4 | 0.7972 | 0.44608 | 1.42471 | 0.28192 | 0.428998462 |
| stage_pN1 | 0.99464 | 0.66923 | 1.47827 | 0.1924 | 0.977941198 |
| stage_pN2 | 0.7862 | 0.50506 | 1.22383 | 0.21487 | 0.273564423 |
| Age_At_Sample_date | 1.00921 | 0.99396 | 1.0247 | 0.0074 | 0.226420372 |
| R = -0.3061387 |  |  |  |  |  |
| **Creatinine** |  |  |  |  |  |
| Term | Slope | 2.5 % | 97.5 % | Standard error | p value |
| (Intercept) | 14.43821 | 1.35736 | 153.57889 | 1.14557 | 0.028504304 |
| log10 (Laboratory test result) | 1.53816 | 0.42842 | 5.52247 | 0.61933 | 0.493577838 |
| stage_pT3 | 0.83198 | 0.47984 | 1.44254 | 0.26666 | 0.49692599 |
| stage_pT4 | 0.78832 | 0.40255 | 1.54377 | 0.32564 | 0.472195701 |
| stage_pN1 | 0.964 | 0.623 | 1.49165 | 0.21151 | 0.863837426 |
| stage_pN2 | 0.78343 | 0.47313 | 1.29723 | 0.24435 | 0.327813986 |
| Age_At_Sample_date | 1.00259 | 0.98587 | 1.01959 | 0.00815 | 0.753628886 |
| R = 0.2374093 |  |  |  |  |  |
| **eGFR** |  |  |  |  |  |
| Term | Slope | 2.5 % | 97.5 % | Standard error | p value |
| (Intercept) | 45.41704 | 1.83173 | 1126.09547 | 1.55561 | 0.0218141 |
| log10 (Laboratory test result) | 0.88099 | 0.22559 | 3.44055 | 0.66008 | 0.849390088 |
| stage_pT3 | 0.85676 | 0.52386 | 1.40123 | 0.23836 | 0.522765972 |
| stage_pT4 | 0.80608 | 0.44856 | 1.44854 | 0.28399 | 0.455194629 |
| stage_pN1 | 0.88388 | 0.59462 | 1.31386 | 0.19206 | 0.526531507 |
| stage_pN2 | 0.66158 | 0.42155 | 1.0383 | 0.21837 | 0.070645412 |
| Age_At_Sample_date | 1.00176 | 0.98493 | 1.01888 | 0.00821 | 0.831749581 |
| R = -0.2136042 |  |  |  |  |  |
| **Bilirubin** |  |  |  |  |  |
| Term | Slope | 2.5 % | 97.5 % | Standard error | p value |
| (Intercept) | 27.63434 | 8.26827 | 92.36 | 0.55853 | 4.88386E-05 |
| log10 (Laboratory test result) | 1.9002 | 0.76672 | 4.70935 | 0.42011 | 0.150455782 |
| stage_pT3 | 0.61564 | 0.35108 | 1.07957 | 0.25998 | 0.08477938 |
| stage_pT4 | 0.59624 | 0.30859 | 1.15203 | 0.30488 | 0.113656766 |
| stage_pN1 | 1.26237 | 0.77012 | 2.06925 | 0.22876 | 0.327018145 |
| stage_pN2 | 1.02585 | 0.58418 | 1.80144 | 0.26063 | 0.923484475 |
| Age_At_Sample_date | 0.99763 | 0.98294 | 1.01255 | 0.00687 | 0.735789789 |
| R = 0.3197274 |  |  |  |  |  |
| **Alkaline phosphatase** |  |  |  |  |  |
| Term | Slope | 2.5 % | 97.5 % | Standard error | p value |
| (Intercept) | 11.65178 | 0.73983 | 183.50764 | 1.25253 | 0.075762511 |
| log10 (Laboratory test result) | 2.23626 | 0.55043 | 9.08545 | 0.63693 | 0.232506356 |
| stage_pT3 | 0.72898 | 0.45013 | 1.1806 | 0.21905 | 0.17686878 |
| stage_pT4 | 0.7515 | 0.38087 | 1.48277 | 0.30877 | 0.374689797 |
| stage_pN1 | 0.90961 | 0.58865 | 1.40557 | 0.19772 | 0.641211515 |
| stage_pN2 | 0.81566 | 0.51325 | 1.29625 | 0.21047 | 0.353781618 |
| Age_At_Sample_date | 0.99543 | 0.9811 | 1.00996 | 0.00658 | 0.500787712 |
| R = 0.2338088 |  |  |  |  |  |
| **Alanine transaminase** |  |  |  |  |  |
| Term | Slope | 2.5 % | 97.5 % | Standard error | p value |
| (Intercept) | 54.3978 | 5.65961 | 522.84863 | 1.02816 | 0.00253323 |
| log10 (Laboratory test result) | 0.93388 | 0.30937 | 2.81905 | 0.50196 | 0.894068227 |
| stage_pT3 | 0.86342 | 0.50169 | 1.48597 | 0.24667 | 0.56367068 |
| stage_pT4 | 1.02928 | 0.52219 | 2.0288 | 0.30831 | 0.927108907 |
| stage_pN1 | 0.99668 | 0.62439 | 1.59094 | 0.21247 | 0.987793956 |
| stage_pN2 | 0.77775 | 0.48952 | 1.23567 | 0.21035 | 0.257231907 |
| Age_At_Sample_date | 0.994 | 0.97776 | 1.0105 | 0.00748 | 0.43788758 |
| R = -0.07001399 |  |  |  |  |  |
| **Low versus high eGFR** |  |  |  |  |  |
| Term | OR | 2.5 % | 97.5 % | Standard error | p value |
| (Intercept) | 0 | 0 | 0.00001 | 2.02232 | 8.60518E-15 |
| log10(cfDNA_GE_p_mL) | 1.37956 | 0.68027 | 2.76986 | 0.35733 | 0.367873311 |
| stage_pT3 | 0.62506 | 0.31584 | 1.16559 | 0.33098 | 0.155683545 |
| stage_pT4 | 0.3374 | 0.09171 | 0.96182 | 0.58718 | 0.064264146 |
| stage_pN1 | 5.41077 | 1.03473 | 99.87553 | 1.05355 | 0.109030186 |
| stage_pN2 | 9.71076 | 1.60676 | 188.41072 | 1.10313 | 0.039329634 |
| Age_At_Sample_date | 1.16262 | 1.12393 | 1.20607 | 0.01797 | 5.00987E-17 |
| AUC = 0.0012 |  |  |  |  |  |
