# Supplementary material for: The role of renal and liver function in clinical ctDNA testing
Source: PLoS One. 2025 Feb 25;20(2):e0319194. doi: 10.1371/journal.pone.0319194 (PMC11856342; doi:10.1371/journal.pone.0319194)
Supplement: Attachment — Submitted filename: Response to Reviewers.docx [file pone.0319194.s010.docx]

**Author Response Letter for revision of manuscript ID PONE-D-24-32619**

Elingarami Sauli, PhD

Academic Editor
PLOS One

2024-10-09

Subject: Submission of revised manuscript Ref. No.: PONE-D-24-32619 entitled “The role of renal and liver function in clinical ctDNA testing”.

Dear Dr Elingarami Sauli

Thank you for the opportunity to revise our paper. The thoughtful and thorough review of our manuscript is appreciated. We extend our gratitude to the reviewers for their input and clarifying questions, which have contributed to strengthening the manuscript. We have carefully reviewed and revised our paper in accordance with the provided comments and have provided a point-by-point response letter below. All references to lines of text follow the updated numbering after revisions in the “tracked changes” version of the manuscript.

We hope you will find the revised manuscript suitable for publication and look forward to hearing from you in due course.

Sincerely,

Claus Lindbjerg Andersen, M.Sc., Ph.D.
Professor, Group Leader, Colorectal Cancer Research Group
Department for Molecular Medicine (MOMA), Aarhus University Hospital

**COMMENTS TO THE AUTHOR**

**Reviewer #1**: The authors investigated whether cfDNA and ctDNA levels are influenced by impaired renal or liver function in patients undergoing curative-intent surgery for early-stage colorectal cancer. Their findings suggest that cfDNA and ctDNA levels remain stable across a broad spectrum of renal and liver function metrics.

However, several important limitations should be considered:

1. The study is focused on pre-operative cfDNA/ctDNA levels, but in clinical practice, post-operative ctDNA is more relevant. It cannot be assumed that post-operative cfDNA/ctDNA levels would remain unaffected by varying degrees of renal or liver function, particularly given the additional variable of DNA fragments released during surgery, which could influence ctDNA clearance in patients with impaired renal or liver function.

Author Response: We appreciate this input from the reviewer. We agree that it is not possible to conclude that post-operative ctDNA levels are unaffected by the liver and renal function of the patients in this cohort. However, we believe that the complexity of the postoperative treatment regime makes it infeasible to draw conclusions about postoperative ctDNA levels with registry data. See our answer to comment number 2 from reviewer #2 for additional details.

2. The method used to calculate eGFR is not clearly described, raising concerns about the accuracy and reliability of the renal function assessment.

Author Response: We are thankful to the reviewer for this vigilant observation. The eGFR results were requisitioned from the regional databases of hospital laboratories using nationally standardized testing methodology. We have added a translated description of the eGFR assessment methodology and eGFR calculation formula in supporting information and revised the manuscript to refer to this [line 108. S1 File].

*“In this study, we define a 30-day preoperative eGFR as the mean of all eGFR measurements conducted in the month leading up to the operation. The formulas used by the regional hospitals to calculate eGFR is available in S1 File.”*

*For ease of the reviewer we have also added them here:*

# Female

P-Creatinine ≤62 µmol/l:

eGFR = 144 x (P-Creatinine /(0.7 x 88.4))-0.329 x 0.993age

P-Creatinine > 62 µmol/l:

eGFR = 144 x (P-Creatinine /(0.7 x 88.4))-1.209 x 0.993age

# Male

P-Creatinine ≤80 µmol/l:

eGFR = 141 x (P-Creatinine /(0.9 x 88.4))-0.411 x 0.993age

P-Creatinine > 80 µmol/l:

eGFR = 141 x (P-Creatinine /(0.9 x 88.4))-1.209 x 0.993age

3. The manuscript does not clarify whether patients had chronic kidney disease, defined as kidney damage or decreased kidney function persisting for three or more months. A single instance of slightly elevated creatinine does not accurately reflect true renal function.

Author Response: We thank the reviewer for bringing this lack of clarity to our attention. We have added a description of sample availability to the methods section [lines 106-112].

*“In this study, we define a 30-day preoperative eGFR as the mean of all eGFR measurements conducted in the month leading up to the operation. The formulas used by the regional hospitals to calculate eGFR is available in S1 File. The median number of eGFR measurements per patient in the month up to operation was 2 (IQR 1-3). The 30-day preoperative eGFR was used to define whether a patient had low renal function or normal renal function. In accordance with Kidney Disease: Improving Global Outcomes (KDGIO) guidelines (https://kdigo.org) patients were categorized as having low renal function if their average eGFR was below 60 mL/min/1.73m^2^.*

We acknowledge the reviewer’s concern in relation to assessing renal function based on a single eGRF measurement. The majority of patients had two or more eGFR measurements made in the 30-day timeframe before operation (n = 300, 62.4%). While we agree that the 30-day eGFR assessments based on a single measurement are less robust, we still believe that the affected patients contribute with important information. Furthermore, as the renal and fluid status measurements conducted in the run-up to operation were made at the treating clinician’s discretion, excluding patients with a single measurement would exacerbate the problem of confounding by indication. Due to these reasons, we abstain from excluding the patients.

In light of the points raised by the reviewer, we have added a section in the discussion to highlight this variance in sample numbers more clearly [line 223-229].

*“Our study focused on laboratory test results in the short 30-day period before curative intended surgery as this period is the standard length of the Danish preoperative CRC cancer work-up program. However, this has the limitation of not meeting KDGIOs recommended 3-month sampling period for assessing long-term renal function. While most of our patients had multiple eGFR measurements in the 30-day period, a minor fraction had just a single eGFR measurement (n = 181 37.6%). These singular measurements provide important information, yet will not account for the patient’s biological variance in eGFR.”*

4. Figure 1 indicates that only a small number of patients had creatinine levels above the normal reference range or an eGFR below the normal reference range. Drawing broad conclusions from such limited data may not be justified. The same concern applies to liver function tests, particularly serum bilirubin levels.

Author Response: We agree with the reviewer that our study offers limited information on patients with extremely low renal and liver function. However, it is highly relevant for the cfDNA/ctDNA community to get a better understanding of the relationship between the natural variation in renal function of CRC patients eligible for surgery and ctDNA test results. The cohort of this study is an unselected representative group of stage I-III CRC patients who are undergoing curative intended surgery. It would be possible to conduct a similar study on patients with end-stage renal and liver failure, but it would offer no actionable information for cancer patients undergoing curative intended treatments, as patients with extremely low renal and liver function are not candidates for surgery. We now address this important topic in the discussion section. [lines 202-204 and lines 236-237]

*“Patients with much lower renal and liver function, than those included in this study, can be found in Denmark, but these would very rarely be candidates for curative intended surgery in the Danish healthcare system.”*

*“For now, it is encouraging that abnormal liver and renal function is of no great concern when conducting ctDNA testing, as the ctDNA clearance can be considered robust across a wide range of test results in CRC patients undergoing curative intended treatment.”*

5. It would be beneficial to categorize patients with eGFR below 60 into subgroups based on the severity of renal impairment (e.g., eGFR 45-59, 30-44, etc.) to determine whether ctDNA levels are affected at specific eGFR threshold.

Author Response: We appreciate the reviewer’s warranted interest in this topic. We agree that a subgroup analysis of eGFR measurements is interesting. We originally chose not include these analyses in the manuscript, because there are very few patients in the severely impacted categories. However, based on the reviewer’s feedback we have now included the analyses in the supplementary materials (S1 and S2 Figures).

In summary, this manuscript does not offer any clinically valuable information.

Author Response: We respectfully disagree with this assertion. The field of ctDNA testing is currently undergoing rapid development. Uncovering possible sources off natural variation in ctDNA and cfDNA measurements remains an important concern for the field. Especially as attempts are made at potentiating the ctDNA levels in the bloodstream for sampling purposes.

**Reviewer #2:** The manuscript reports an interesting concept of impact of variation in renal and liver functions on the detection of ctDNA/cfDNA in patients with resectable colorectal cancer. The conclusion is that there is no such effect. I have the following comments.

1. The only major limitation in this study is that the majority of the participants have their renal/liver functions within reference range and hardly any participant has severe renal or liver dysfunction. This should be clearly highlighted as a limitation and reason for contradiction from the the pre-clinical and previously published reports.

Author response: We are thankful for the reviewer’s insightful and relevant suggestion. We do believe that our cohort is well suited to investigate whether ctDNA and cfDNA clearance inhibition occurs naturally in stage I-III CRC patients undergoing curative intended surgery. We agree wholeheartedly that it is important to communicate that our study does not provide much information on whether severe renal and liver dysfunction could inhibit cfDNA and ctDNA clearance. We have amended the discussion section to more clearly address the low prevalence of severe renal and liver function in our cohort. [lines 202-204]

*“Patients with much lower renal and liver function, than those included in this study, can be found in Denmark, but these would very rarely be candidates for curative intended surgery in the Danish healthcare system.”*

2. It appears the bloods samples for ctDNA were taken both pre and post-surgery, but only pre-op data is presented. Did they compare post op ctDNA with post-op renal/liver functions?

Author response: We appreciate the reviewer’s interest in preoperative ctDNA and postoperative renal/liver functions. Our study elects to focus on preoperative laboratory biomarkers and ctDNA tests. Preoperatively, patients have their liver and renal function systematically assessed as part of the clinical workup before surgery. In the postoperative situation, liver and renal function are much less systematic, meaning that indication, intervention, number of measurements and the timing vary considerably. A large fraction of these measurements was made after clinical intervention and outlier values were often quickly normalized. Accordingly, registry data of post-operative clinical renal and liver assessments are not an appropriate data source for making generalizable correlations between cfDNA/ctDNA and renal/liver function. In our view, elucidating the immediate post-operative ctDNA and cfDNA metabolism of cancer patients would require a clinical study specifically designed to prospectively and systematically gather the needed data (liver/renal/cfDNA/ctDNA measurements made at the same time-point), which is beyond the scope of this article.

The reviewer’s comment made us reflect on the methods section describing the blood samples available for ctDNA analysis. [Line 79 in original manuscript]

*“As part of the study, patients had blood collected prior to and after surgery, with the aim to retrospectively measure cfDNA and ctDNA.”*

As our study does not involve the postoperative samples, we have elected to remove the mentioning of these samples to avoid confusion. [Line 74]

**Editorial office revision requests**

1. Please ensure that your manuscript meets PLOS ONE's style requirements, including those for file naming. The PLOS ONE style templates can be found at

https://journals.plos.org/plosone/s/file?id=wjVg/PLOSOne_formatting_sample_main_body.pdf

and

https://journals.plos.org/plosone/s/file?id=ba62/PLOSOne_formatting_sample_title_authors_affiliations.pdf

Author response: We thank the editor for the resources provided and have used them to revise the manuscript to follow PLOS ONE’s style requirements: A list of changes is given below:

1. Edited headings to use sentence case, resized heads to 18pts for level 1 headings and 16pts for level 2 headings. Bolded all headings
2. Revised figure and table citations (example: figure 1 -> Fig 1. Supplementary table S1 -> S1 Table)
3. Revised filenames to follow guidelines
4. Unbolded figure captions except for figure title.
5. Changed title of Supplementary materials to “Supporting information” and updated contents to reflect manuscript edits.
6. Moved “supporting information section” to end of document
7. Removed the title of the “affiliations” section
8. Removed superfluous corresponding author information. Only email is listed now
9. Removed funding and support section
10. Removed declaration of competing interests section

2. Please update your submission to use the PLOS LaTeX template. The template and more information on our requirements for LaTeX submissions can be found at http://journals.plos.org/plosone/s/latex.

Author response: We have elected to resubmit the manuscript in DOCX format. The original PDF submission was a mistake due to misunderstanding the author guidelines. We apologize for the mistake and are grateful for the opportunity to correct it.

3. In the online submission form you indicate that your data is not available for proprietary reasons and have provided a contact point for accessing this data. Please note that your current contact point is a co-author on this manuscript. According to our Data Policy, the contact point must not be an author on the manuscript and must be an institutional contact, ideally not an individual. Please revise your data statement to a non-author institutional point of contact, such as a data access or ethics committee, and send this to us via return email. Please also include contact information for the third party organization, and please include the full citation of where the data can be found.

Author response: We have edited the manuscript so that the contact point is The Department of Molecular Medicine at moma@rm.dk and included a description of how to use the mail to request data access.

*“Because the data contains sensitive personal patient information, it cannot be made publicly available. Access to data requires that the data requestor (legal entity) enter into Collaboration and Data Processing Agreements, with the Central Denmark Region (the legal entity controlling and responsible for the data). Inquiries for access can be addressed to the Data Access Committee at Department of Molecular Medicine, Aarhus University Hospital (contact via moma@rm.dk).”*
